# Supplementary material for: Resolving the full spectrum of human genome variation using Linked-Reads
Source: Genome Res. 2019 Apr;29(4):635–45. doi: 10.1101/gr.234443.118 (PMC6442396; doi:10.1101/gr.234443.118)
Supplement: Supplemental Material [file supp_gr.234443.118_Supplemental_longranger-2.2.zip › longranger-2.2.tar.bz2!/lr2.2-public/lib/stan/cmdstan/stan/doc/api/html/agrad_2rev_2matrix_2dot__product_8hpp_source.html]

Stan: src/stan/agrad/rev/matrix/dot\_product.hpp Source File


|  |  |
| --- | --- |
|  | Stan  2.5.0  probability, sampling & optimization |


- Main Page
- Related Pages
- Namespaces
- Classes
- Files

- File List
- File Members

All Classes Namespaces Files Functions Variables Typedefs Enumerations Enumerator Friends Macros Pages

- src
- stan
- agrad
- rev
- matrix

dot\_product.hpp

Go to the documentation of this file.

1 #ifndef STAN\_\_AGRAD\_\_REV\_\_MATRIX\_\_DOT\_PRODUCT\_HPP

2 #define STAN\_\_AGRAD\_\_REV\_\_MATRIX\_\_DOT\_PRODUCT\_HPP

3

4 #include <vector>

5 #include <boost/utility/enable\_if.hpp>

6 #include <boost/type\_traits.hpp>

7 #include <stan/math/matrix/Eigen.hpp>

8 #include <stan/math/matrix/typedefs.hpp>

9 #include <stan/math/error\_handling/matrix/check\_vector.hpp>

10 #include <stan/math/error\_handling/matrix/check\_matching\_sizes.hpp>

11 #include <stan/math/functions/value\_of.hpp>

12 #include <stan/agrad/rev/var.hpp>

13 #include <stan/agrad/rev/vari.hpp>

14 #include <stan/agrad/rev/matrix/typedefs.hpp>

15 #include <stan/agrad/rev/functions/value\_of.hpp>

16

17 namespace stan {

18  namespace agrad {

19

20  namespace {

21  template<typename T>

22  struct dot\_product\_store\_type;

23

24  template<>

25  struct dot\_product\_store\_type<var> {

26  typedef vari\*\* type;

27  };

28

29  template<>

30  struct dot\_product\_store\_type<double> {

31  typedef double\* type;

32  };

33

34  template<typename T1, typename T2>

35  class dot\_product\_vari : public vari {

36  protected:

37  typename dot\_product\_store\_type<T1>::type v1\_;

38  typename dot\_product\_store\_type<T2>::type v2\_;

39  size\_t length\_;

40

41  inline static double var\_dot(vari\*\* v1, vari\*\* v2,

42  size\_t length) {

43  Eigen::VectorXd vd1(length), vd2(length);

44  for (size\_t i = 0; i < length; i++) {

45  vd1[i] = v1[i]->val\_;

46  vd2[i] = v2[i]->val\_;

47  }

48  return vd1.dot(vd2);

49  }

50

51  inline static double var\_dot(const T1\* v1, const T2\* v2,

52  size\_t length) {

53  using stan::math::value\_of;

54  Eigen::VectorXd vd1(length), vd2(length);

55  for (size\_t i = 0; i < length; i++) {

56  vd1[i] = value\_of(v1[i]);

57  vd2[i] = value\_of(v2[i]);

58  }

59  return vd1.dot(vd2);

60  }

61

62  template<typename Derived1,typename Derived2>

63  inline static double var\_dot(const Eigen::DenseBase<Derived1> &v1,

64  const Eigen::DenseBase<Derived2> &v2) {

65  using stan::agrad::value\_of;

66  using stan::math::value\_of;

67  Eigen::VectorXd vd1(v1.size()), vd2(v1.size());

68  for (int i = 0; i < v1.size(); i++) {

69  vd1[i] = value\_of(v1[i]);

70  vd2[i] = value\_of(v2[i]);

71  }

72  return vd1.dot(vd2);

73  }

74  inline void chain(vari\*\* v1, vari\*\* v2) {

75  for (size\_t i = 0; i < length\_; i++) {

76  v1[i]->adj\_ += adj\_ \* v2\_[i]->val\_;

77  v2[i]->adj\_ += adj\_ \* v1\_[i]->val\_;

78  }

79  }

80  inline void chain(double\* v1, vari\*\* v2) {

81  for (size\_t i = 0; i < length\_; i++) {

82  v2[i]->adj\_ += adj\_ \* v1\_[i];

83  }

84  }

85  inline void chain(vari\*\* v1, double\* v2) {

86  for (size\_t i = 0; i < length\_; i++) {

87  v1[i]->adj\_ += adj\_ \* v2\_[i];

88  }

89  }

90  inline void initialize(vari\*\* &mem\_v, const var \*inv, vari \*\*shared = NULL) {

91  if (shared == NULL) {

92  mem\_v = (vari\*\*)memalloc\_.alloc(length\_\*sizeof(vari\*));

93  for (size\_t i = 0; i < length\_; i++)

94  mem\_v[i] = inv[i].vi\_;

95  }

96  else {

97  mem\_v = shared;

98  }

99  }

100  template<typename Derived>

101  inline void initialize(vari\*\* &mem\_v, const Eigen::DenseBase<Derived> &inv, vari \*\*shared = NULL) {

102  if (shared == NULL) {

103  mem\_v = (vari\*\*)memalloc\_.alloc(length\_\*sizeof(vari\*));

104  for (size\_t i = 0; i < length\_; i++)

105  mem\_v[i] = inv(i).vi\_;

106  }

107  else {

108  mem\_v = shared;

109  }

110  }

111

112  inline void initialize(double\* &mem\_d, const double \*ind, double \*shared = NULL) {

113  if (shared == NULL) {

114  mem\_d = (double\*)memalloc\_.alloc(length\_\*sizeof(double));

115  for (size\_t i = 0; i < length\_; i++)

116  mem\_d[i] = ind[i];

117  }

118  else {

119  mem\_d = shared;

120  }

121  }

122  template<typename Derived>

123  inline void initialize(double\* &mem\_d, const Eigen::DenseBase<Derived> &ind, double \*shared = NULL) {

124  if (shared == NULL) {

125  mem\_d = (double\*)memalloc\_.alloc(length\_\*sizeof(double));

126  for (size\_t i = 0; i < length\_; i++)

127  mem\_d[i] = ind(i);

128  }

129  else {

130  mem\_d = shared;

131  }

132  }

133

134  public:

135  dot\_product\_vari(typename dot\_product\_store\_type<T1>::type v1,

136  typename dot\_product\_store\_type<T2>::type v2,

137  size\_t length)

138  : vari(var\_dot(v1,v2,length)), v1\_(v1), v2\_(v2), length\_(length) {}

139

140  dot\_product\_vari(const T1\* v1, const T2\* v2, size\_t length,

141  dot\_product\_vari<T1,T2>\* shared\_v1 = NULL,

142  dot\_product\_vari<T1,T2>\* shared\_v2 = NULL) :

143  vari(var\_dot(v1, v2, length)), length\_(length) {

144  if (shared\_v1 == NULL) {

145  initialize(v1\_,v1);

146  }

147  else {

148  initialize(v1\_,v1,shared\_v1->v1\_);

149  }

150  if (shared\_v2 == NULL) {

151  initialize(v2\_,v2);

152  }

153  else {

154  initialize(v2\_,v2,shared\_v2->v2\_);

155  }

156  }

157  template<typename Derived1,typename Derived2>

158  dot\_product\_vari(const Eigen::DenseBase<Derived1> &v1,

159  const Eigen::DenseBase<Derived2> &v2,

160  dot\_product\_vari<T1,T2>\* shared\_v1 = NULL,

161  dot\_product\_vari<T1,T2>\* shared\_v2 = NULL) :

162  vari(var\_dot(v1, v2)), length\_(v1.size()) {

163  if (shared\_v1 == NULL) {

164  initialize(v1\_,v1);

165  }

166  else {

167  initialize(v1\_,v1,shared\_v1->v1\_);

168  }

169  if (shared\_v2 == NULL) {

170  initialize(v2\_,v2);

171  }

172  else {

173  initialize(v2\_,v2,shared\_v2->v2\_);

174  }

175  }

176  template<int R1,int C1,int R2,int C2>

177  dot\_product\_vari(const Eigen::Matrix<T1,R1,C1> &v1,

178  const Eigen::Matrix<T2,R2,C2> &v2,

179  dot\_product\_vari<T1,T2>\* shared\_v1 = NULL,

180  dot\_product\_vari<T1,T2>\* shared\_v2 = NULL) :

181  vari(var\_dot(v1, v2)), length\_(v1.size()) {

182  if (shared\_v1 == NULL) {

183  initialize(v1\_,v1);

184  }

185  else {

186  initialize(v1\_,v1,shared\_v1->v1\_);

187  }

188  if (shared\_v2 == NULL) {

189  initialize(v2\_,v2);

190  }

191  else {

192  initialize(v2\_,v2,shared\_v2->v2\_);

193  }

194  }

195  virtual void chain() {

196  chain(v1\_,v2\_);

197  }

198  };

199  }

200

209  template<typename T1, int R1, int C1, typename T2, int R2, int C2>

210  inline

211  typename boost::enable\_if\_c<boost::is\_same<T1,var>::value ||

212  boost::is\_same<T2,var>::value, var>::type

213  dot\_product(const Eigen::Matrix<T1, R1, C1>& v1,

214  const Eigen::Matrix<T2, R2, C2>& v2) {

215  stan::math::check\_vector("dot\_product(%1%)",v1,"v1",(double\*)0);

216  stan::math::check\_vector("dot\_product(%1%)",v2,"v2",(double\*)0);

217  stan::math::check\_matching\_sizes("dot\_product(%1%)",v1,"v1",

218  v2,"v2",(double\*)0);

219  return var(new dot\_product\_vari<T1,T2>(v1,v2));

220  }

229  template<typename T1, typename T2>

230  inline

231  typename boost::enable\_if\_c<boost::is\_same<T1,var>::value ||

232  boost::is\_same<T2,var>::value, var>::type

233  dot\_product(const T1\* v1, const T2\* v2, size\_t length) {

234  return var(new dot\_product\_vari<T1,T2>(v1, v2, length));

235  }

236

245  template<typename T1, typename T2>

246  inline

247  typename boost::enable\_if\_c<boost::is\_same<T1,var>::value ||

248  boost::is\_same<T2,var>::value, var>::type

249  dot\_product(const std::vector<T1>& v1,

250  const std::vector<T2>& v2) {

251  stan::math::check\_matching\_sizes("dot\_product(%1%)",v1,"v1",

252  v2,"v2",(double\*)0);

253  return var(new dot\_product\_vari<T1,T2>(&v1[0], &v2[0], v1.size()));

254  }

255

256  template<typename T1, int R1, int C1, typename T2, int R2, int C2>

257  inline

258  typename boost::enable\_if\_c<boost::is\_same<T1,var>::value ||

259  boost::is\_same<T2,var>::value,

260  Eigen::Matrix<var, 1, C1> >::type

261  columns\_dot\_product(const Eigen::Matrix<T1, R1, C1>& v1,

262  const Eigen::Matrix<T2, R2, C2>& v2) {

263  stan::math::check\_matching\_sizes("dot\_product(%1%)",v1,"v1",

264  v2,"v2",(double\*)0);

265  Eigen::Matrix<var, 1, C1> ret(1,v1.cols());

266  for (size\_type j = 0; j < v1.cols(); ++j) {

267  ret(j) = var(new dot\_product\_vari<T1,T2>(v1.col(j),v2.col(j)));

268  }

269  return ret;

270  }

271

272  template<typename T1, int R1, int C1, typename T2, int R2, int C2>

273  inline

274  typename boost::enable\_if\_c<boost::is\_same<T1,var>::value ||

275  boost::is\_same<T2,var>::value,

276  Eigen::Matrix<var, R1, 1> >::type

277  rows\_dot\_product(const Eigen::Matrix<T1, R1, C1>& v1,

278  const Eigen::Matrix<T2, R2, C2>& v2) {

279  stan::math::check\_matching\_sizes("dot\_product(%1%)",v1,"v1",

280  v2,"v2",(double\*)0);

281  Eigen::Matrix<var, R1, 1> ret(v1.rows(),1);

282  for (size\_type j = 0; j < v1.rows(); ++j) {

283  ret(j) = var(new dot\_product\_vari<T1,T2>(v1.row(j),v2.row(j)));

284  }

285  return ret;

286  }

287  }

288 }

289 #endif

vari.hpp

typedefs.hpp

typedefs.hpp

stan::length

size\_t length(const T &)

**Definition:** traits.hpp:159

stan::agrad::memalloc\_

memory::stack\_alloc memalloc\_

**Definition:** var\_stack.cpp:16

stan::math::check\_vector

bool check\_vector(const char \*function, const Eigen::Matrix< T, R, C > &x, const char \*name, T\_result \*result)

**Definition:** check\_vector.hpp:14

check\_matching\_sizes.hpp

stan::agrad::value\_of

T value\_of(const fvar< T > &v)

Return the value of the specified variable.

**Definition:** value\_of.hpp:16

stan::math::initialize

void initialize(T &x, const T &v)

**Definition:** initialize.hpp:17

stan::agrad::inv

fvar< T > inv(const fvar< T > &x)

**Definition:** inv.hpp:15

stan::math::value\_of

double value\_of(const T x)

Return the value of the specified scalar argument converted to a double value.

**Definition:** value\_of.hpp:24

v2\_

dot\_product\_store\_type< T2 >::type v2\_

**Definition:** dot\_product.hpp:38

stan::agrad::size\_type

Eigen::Matrix< double, Eigen::Dynamic, Eigen::Dynamic >::Index size\_type

**Definition:** typedefs.hpp:14

stan::agrad::dot\_product

fvar< T > dot\_product(const Eigen::Matrix< fvar< T >, R1, C1 > &v1, const Eigen::Matrix< fvar< T >, R2, C2 > &v2)

**Definition:** dot\_product.hpp:20

var.hpp

Eigen.hpp

stan::memory::stack\_alloc::alloc

void \* alloc(size\_t len)

Return a newly allocated block of memory of the appropriate size managed by the stack allocator...

**Definition:** stack\_alloc.hpp:168

check\_vector.hpp

v1\_

dot\_product\_store\_type< T1 >::type v1\_

**Definition:** dot\_product.hpp:37

stan::agrad::var

Independent (input) and dependent (output) variables for gradients.

**Definition:** var.hpp:27

stan::math::size

int size(const std::vector< T > &x)

**Definition:** size.hpp:11

value\_of.hpp

stan::agrad::columns\_dot\_product

Eigen::Matrix< fvar< T >, 1, C1 > columns\_dot\_product(const Eigen::Matrix< fvar< T >, R1, C1 > &v1, const Eigen::Matrix< fvar< T >, R2, C2 > &v2)

**Definition:** columns\_dot\_product.hpp:18

stan::math::check\_matching\_sizes

bool check\_matching\_sizes(const char \*function, const T\_y1 &y1, const char \*name1, const T\_y2 &y2, const char \*name2, T\_result \*result)

**Definition:** check\_matching\_sizes.hpp:15

length\_

size\_t length\_

**Definition:** dot\_product.hpp:39

value\_of.hpp

stan::agrad::rows\_dot\_product

Eigen::Matrix< fvar< T >, R1, 1 > rows\_dot\_product(const Eigen::Matrix< fvar< T >, R1, C1 > &v1, const Eigen::Matrix< fvar< T >, R2, C2 > &v2)

**Definition:** rows\_dot\_product.hpp:19


---

[ Stan Home Page ]

*© 2011–2014,
Stan Development Team.*
